# Structure and calcium-binding studies of calmodulin-like domain of human non-muscle α-actinin-1

**DOI:** 10.1038/srep27383

**Published:** 2016-06-07

**Authors:** Sara Drmota Prebil, Urška Slapšak, Miha Pavšič, Gregor Ilc, Vid Puž, Euripedes de Almeida Ribeiro, Dorothea Anrather, Markus Hartl, Lars Backman, Janez Plavec, Brigita Lenarčič, Kristina Djinović-Carugo

**Affiliations:** 1 Department of Chemistry and Biochemistry, Faculty of Chemistry and Chemical Technology, University of Ljubljana, Večna pot 113, SI-1000 Ljubljana, Slovenia; 2 Slovenian NMR Centre, National Institute of Chemistry, Hajdrihova 19, SI-1000 Ljubljana, Slovenia; 3 EN-FIST Centre of Excellence, Trg Osvobodilne fronte 13, SI-1000 Ljubljana, Slovenia; 4 Department of Structural and Computational Biology, Max F. Perutz Laboratories (MFPL), University of Vienna, Campus Vienna Biocenter 5, A-1030 Vienna, Austria; 5 Mass Spectrometry Service Facility, Max F. Perutz Laboratories (MFPL), University of Vienna, Vienna Biocenter (VBC), Dr. Bohr-Gasse 3, A-1030 Vienna, Austria; 6 Department of Chemistry, Umeå University, Linnaeus väg 10, SE-90187 Umeå, Sweden; 7 Department of Biochemistry, Molecular Biology and Structural Biology, Jožef Stefan Institute, Jamova 39,SI-1000 Ljubljana, Slovenia

## Abstract

The activity of several cytosolic proteins critically depends on the concentration of calcium ions. One important intracellular calcium-sensing protein is α-actinin-1, the major actin crosslinking protein in focal adhesions and stress fibers. The actin crosslinking activity of α-actinin-1 has been proposed to be negatively regulated by calcium, but the underlying molecular mechanisms are poorly understood. To address this, we determined the first high-resolution NMR structure of its functional calmodulin-like domain (CaMD) in calcium-bound and calcium-free form. These structures reveal that in the absence of calcium, CaMD displays a conformationally flexible ensemble that undergoes a structural change upon calcium binding, leading to limited rotation of the N- and C-terminal lobes around the connecting linker and consequent stabilization of the calcium-loaded structure. Mutagenesis experiments, coupled with mass-spectrometry and isothermal calorimetry data designed to validate the calcium binding stoichiometry and binding site, showed that human non-muscle α-actinin-1 binds a single calcium ion within the N-terminal lobe. Finally, based on our structural data and analogy with other α-actinins, we provide a structural model of regulation of the actin crosslinking activity of α-actinin-1 where calcium induced structural stabilisation causes fastening of the juxtaposed actin binding domain, leading to impaired capacity to crosslink actin.

The actin cytoskeleton plays an essential role in many important cellular processes, such as muscle cell contraction, motility, signalling, intracellular traffic, and maintenance of cell shape and stability. The central components of the actin cytoskeleton are actin filaments, which are further equipped with different proteins giving them various functionalities. α-actinins are one of the major actin cross-linking proteins found in virtually all cell types and are the ancestral proteins of a larger family that includes spectrin, dystrophin and utrophin^1^. Four isoforms of human α-actinins have been identified: the calcium-insensitive muscle actinins (isoforms 2 and 3), which crosslink actin filaments in sarcomere-delimiting Z-disk complexes, and calcium-sensitive non-muscle isoforms (isoforms 1 and 4). The non-muscle isoforms are found in all types of cells where they crosslink and organize actin filaments into two general types of structures: three-dimensional networks, where the actin filaments are oriented in various directions, and bundles of tightly packed parallel actin filaments^2^,^3^. Bundles function as scaffolds that support or stabilize cellular structures such as focal adhesion contacts, cell protrusions, and stress fibers^2^,^4^,^5^. For example, it was shown that dorsal stress fibres require α-actinin-1 and that their loss in α-actinin-1 depleted cells results in defective maturation of the leading edge in focal adhesions^6^.

The actin crosslinking function of all α-actinin isoforms is determined by its overall domain organization (Fig. 1), where the two actin-binding domains (ABD, composed of calponin homology domains CH1 and CH2) are located on opposite ends of the antiparallel dimer, stabilized by extensive subunit interactions between the central rod domain, which is composed of four spectrin repeats (SR)^7^,^8^,^9^. The ABD is connected to the rod domain via a flexible neck region. The calmodulin-like domain (CaMD) of the opposite subunit is located in close proximity, where it regulates the actin-crosslinking activity of α-actinin^9^. Non-muscle α-actinins are inhibited by calcium from cross-linking of F-actin and to a lesser extent from binding to F-actin^10^.

CaMD of calcium-sensing proteins is composed of an N- and a C-terminal lobe, each comprised of a pair of interacting EF hands (EF1-2 and EF3-4) (refs 11,12). Binding of Ca^2+^ stabilizes a partially unstructured loop region connecting the two helices within the EF hand and reduces the internal mobility of all helices within the domain. At the same time, partner helices within each EF hand are re-oriented from nearly antiparallel to an almost perpendicular orientation. This results in exposure of large hydrophobic patches on the surface of each lobe (closed-to-open transition) and increased affinity toward its interaction partners^12^.

Some insight into the calcium regulation mechanism of non-muscle α-actinin is provided by a model of the ternary complex between ABD, adjacent helical neck region, and the CaMD of α-actinin-4 (ref. 13). Mutagenesis experiments showed that complex disruption enhances binding of α-actinin to actin^13^. Furthermore, these results suggest that calcium binding to EF1-2 is necessary for the interaction of EF3-4 with the neck region. The important role of EF3-4 and the neck region interaction is also supported by the recently described regulation mechanism of muscle α-actinin-2 (ref. 14). Here, in contrast to α-actinin-4, binding of PIP_2_ disrupts the otherwise stable interaction between EF3-4 and the neck region, thereby enabling binding of α-actinin-2 to titin (via EF3-4) (ref. 14). Since α-actinin isoforms are highly similar, binding of EF3-4 to the neck region could represent a common event in the regulatory mechanism of the actin binding activity of α-actinin. However, to construct a sound model, detailed structural analysis of α-actinin calcium binding and accompanying conformational changes is a prerequisite.

To provide insight into the molecular basis of the calcium regulation of non-muscle α-actinins, we performed detailed structural and biophysical analysis of the CaMD of α-actinin-1. First, we show that CaMD binds only one calcium ion in EF1 of the N-terminal lobe. Next, we report the first high-resolution three-dimensional NMR structures of apo and holo forms (i.e., without and with bound calcium ions) of CaMD which reveal that calcium binding induces structure stabilisation and increases rigidity in the linker between the two lobes of the CaMD. Finally, we constructed a model of Ca^2+^-induced structural changes of α-actinin-1, which are central to its actin-crosslinking activity.

## Results

### CaMD of α-actinin-1 contains only one canonical calcium-binding site

**Figure 2:**
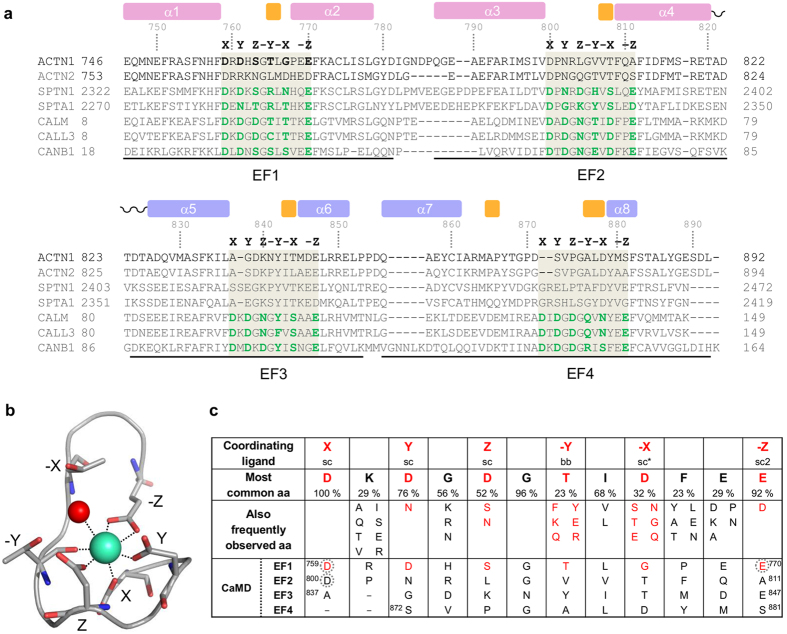
Sequence alignment of α-actinin-1 EF1-4 and calcium-binding domains of selected representative human proteins. (**a**) Both putative and experimentally confirmed calcium-binding motifs are shown as grey rectangles. Residues critical for Ca^2+^ coordination (positions X, Y, Z, -Y, -X, -Z) in EF hands with known calcium-binding activity are shown in green and bold. ACTN1 - α-actinin-1, ACTN2 - α-actinin-2, SPTN1 - α chain of non-erythrocytic spectrin, SPTA1 - α chain of erythrocytic spectrin, CALM - calmodulin, CALL3 - calmodulin-like protein 3, CANB1 - subunit B of type I calcineurin. Alignments were prepared using ClustalW^54^. Residue numbering follows α-actinin-1. α-helices of the apo form are denoted as pink (EF1-2) or violet (EF3-4) rectangles, β-strand secondary structure elements as orange rectangles, and the linker between the two lobes as a curved line. (**b**) Calcium coordination by the canonical EF hand (EF1 of calmodulin, PDB ID 1CLL) illustrating the pentagonal bipyramidal coordination of the Ca^2+^ ion (broken lines). NH groups of coordinating amino acids are indicated in dark blue, oxygen atoms in red, the Ca^2+^ ion in green-cyan and the coordinating water molecule in red. (**c**) Sequence preference of the EF hand loop^55^. Ca^2+^ ligands are indicated by their positions in the coordination sphere (X, Y, Z, -X, -Y and -Z). Coordination occurs via side chains (sc) or through the backbone (bb) of amino acids shown in red. The asterisk highlights the ligand typically provided by a water molecule that is hydrogen-bonded to the side chain of the amino acid at position 9, the label sc2 indicates bidentate ligand. The most common amino acids at each position, with their corresponding percentages of occurrence, and those that occur with a frequency greater than 5% in known EF-loops, are shown^19^. Residues mutated in our study are circled.

To assess the calcium-binding potential of the four putative calcium sites of α-actinin-1 (EF1 to EF4), we compared their sequences to sequences of EF hands from several other calcium-binding proteins, in which the Ca^2+^ ion is coordinated in a canonical pentagonal bipyramidal configuration (Fig. 2a,b).

The EF hand motif consists of two short alpha helices connected by a loop region, which, together with a single water molecule, provide seven ligands coordinating a single Ca^2+^ ion in a pentagonal bipyramidal coordination sphere. The six residues (relative sequence positions 1, 3, 5, 7, 9 and 12) involved in Ca^2+^ binding are denoted X, Y, Z, -Y, -X and -Z^15^,^16^. The invariant bidentate ligand, Glu or Asp, at position -Z (12) provides two oxygens to the Ca^2+^ coordination sphere (Fig. 2b). In calcium-insensitive muscle isoforms of α-actinin, some residues critical for Ca^2+^ coordination are not conserved, explaining their lack of Ca^2+^ binding potential (Fig. 2a)^9^,^17^,^18^.

Comparative inspection of amino acid sequences^16^,^19^ (Fig. 2c) and ScanProsite analysis^20^ shows that in calcium-sensitive non-muscle isoforms of α-actinin, all of the residues required for Ca^2+^ coordination at positions X, Y, Z, -Y, -X and -Z are only present in EF1. This implies that only EF1 is able to bind Ca^2+^, while EF2, which lacks canonical residues at positions Z, -Y and -Z, is not. Additionally, EF3 and EF4 appear to be unable to bind Ca^2+^ due to deletions in the EF-binding loop motifs and lack of key coordinating residues.

In order to experimentally validate the presence of a single calcium-binding site in CaMD of α-actinin-1 and to assess its calcium-binding properties, calcium binding to wild-type and mutant forms of CaMD was compared using native electrospray mass spectrometry (ESI-MS) and isothermal titration calorimetry (ITC). In mutant forms, calcium-binding sites within EF1 (CaMD_D759A and CaMD_E770A) and EF2 (CaMD_D800A) as a negative control were selectively inactivated by introduction of site-specific mutations (Asp or Glu to Ala at X or –Z coordination position).

The proposed single calcium-binding site in CaMD was first addressed by native ESI-MS analysis (see Supplementary Fig. S1). The mass difference for wild-type CaMD and a CaMD_D800A mutant in the presence and absence of calcium can be attributed to a single calcium ion, whereas the mass of the CaMD_D759A mutant corresponds to calcium-free protein under both conditions.

**Figure 3:**
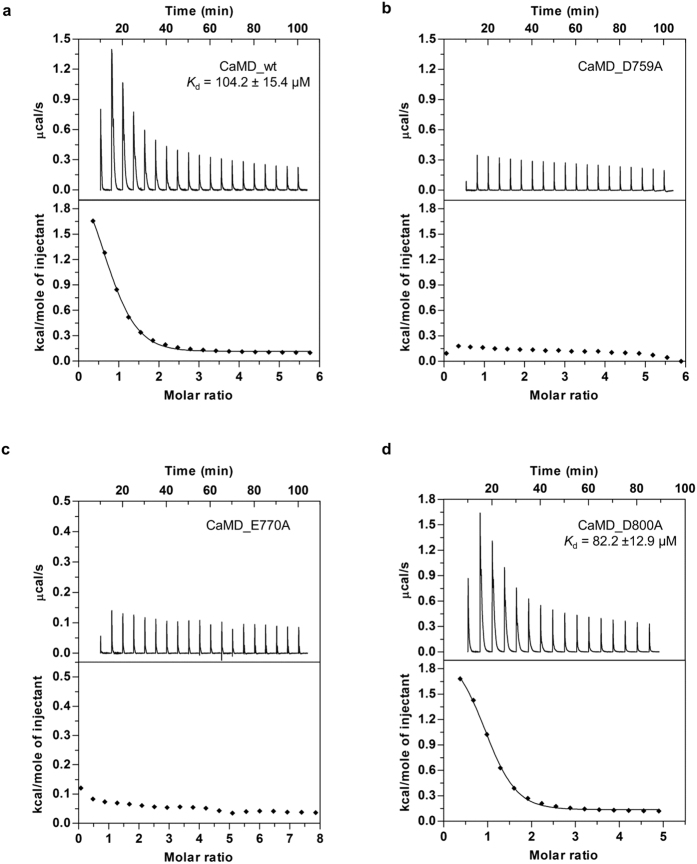
Calcium titration of CaMD as monitored by ITC. (**a**) Wild-type CaMD, (**b**) CaMD_D759A mutant, (**c**) CaMD_E770A mutant, (**d**) CaMD_D800A mutant. The upper panels show measured raw heat changes as a function of time, while the lower panels show integrated heat changes after subtracting the heat of dilution at different Ca^2+^/protein molar ratios. Calculated thermodynamic parameters are shown in Table 1.

Representative isotherms for calcium binding to isolated wild-type CaMD and mutant forms of CaMD are shown in Fig. 3. The thermogram for wild-type CaMD displays a single endothermic event at a molar ratio of *n* = 0.75 ± 0.07 and corresponds to binding of a single calcium ion with a *K*_d_ of 104.2 ± 15.4 μM. The thermogram for CaMD_D759A does not indicate calcium binding. Similarly, no binding was observed for CaMD_E770A. In contrast, the D800A mutation did not have any effect on calcium binding; here, the thermogram closely resembles that obtained for wild-type CaMD.

The thermodynamic parameters of calcium binding calculated from ITC measurements using the wild-type and D800A mutant form of CaMD are listed in Table 1. ITC curves for Ca^2+^ binding to wild-type CaMD and the D800A mutant exhibit an endothermic profile, indicating coupling with a favourable entropic change, thus confirming that EF2 does not bind calcium. Collectively, these results show one calcium-binding site located in EF1 of CaMD and confirm the recently published ITC data indicating that there is only one Ca^2+^ binding site in α-actinin-1 (ref. 21).

### Calcium binding stabilizes the overall structure of α-actinin-1 CaMD

^1^H-^15^N HSQC spectra of double-labeled apo and holo forms of CaMD of α-actinin-1 show good dispersion of cross-peaks, thus enabling detailed structural characterization (see Supplementary Figs S2 and S3). Sequence-specific assignment was performed with the use of standard triple resonance NMR experiments. The high number of NOE distances, torsion angles and RDC restraints obtained, together with the completeness of resonance assignments enabled determination of high-resolution structures of both forms (see Supplementary Table S1).

**Figure 4:**
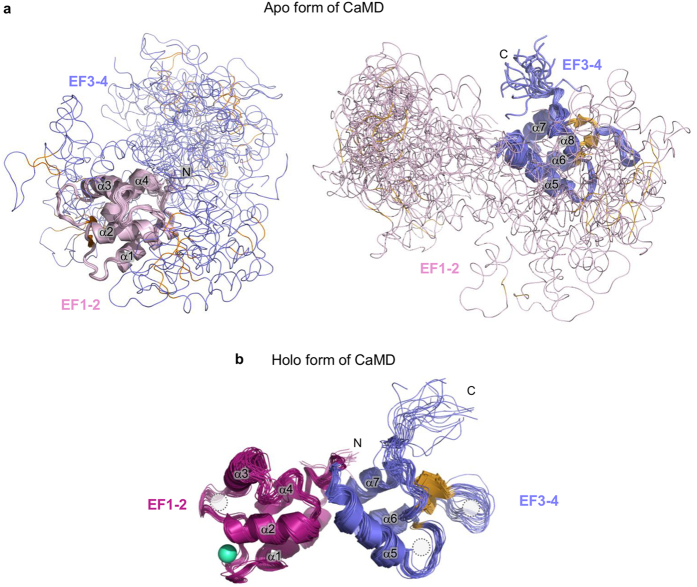
Ensemble of 20 lowest energy structures of the apo and holo forms of CaMD of α-actinin-1. (**a**) Structure of the apo form; superposition of a region comprising residues 743-820 of EF1-2 (left) and 825-892 of EF3-4 (right). EF1-2 is shown in light pink, EF3-4 in violet, and β-strands in orange. (**b**) Structure of the holo form. Color-coding is the same as in (**a**) except for EF1-2, which is shown in magenta. Ca^2+^ ion is depicted as a green-cyan sphere. The three dotted circles mark three EF hand motifs which, do not bind calcium ions (EF2-4).

All EF hands have a typical overall structure composed of two helices connected by a short linker region (α1–α2 for EF1, α3–α4 for EF2, α5−α6 for EF3, and α7−α8 for EF4). In the apo form of CaMD, the individual lobes (EF1-2 and EF3-4) are well defined, with a backbone RMSD of 0.65 and 1.13 Å, respectively. Their relative orientation cannot be unambiguously defined due to the lack of long-range NOE restraints between them (Fig. 4a). This result is in agreement with the NMR structure of calmodulin, which displays high flexibility in solution with vastly different relative orientations between the two domains^12^. On the other hand, the holo form is strikingly different, since the EF1-2 and EF3-4 lobes adopt a well-defined relative orientation (Fig. 4b). The RMSD for all backbone atoms of the holo form of CaMD of α-actinin-1 is 1.13 Å.

Structural analysis of both apo and holo forms suggest that calcium binding locks the relative orientation of the N- and C-terminal lobes, caused by establishment of a series of stabilising interactions mediated by the linker (aa 820 – 825), which displays a single conformation in the holo form.

**Figure 5:**
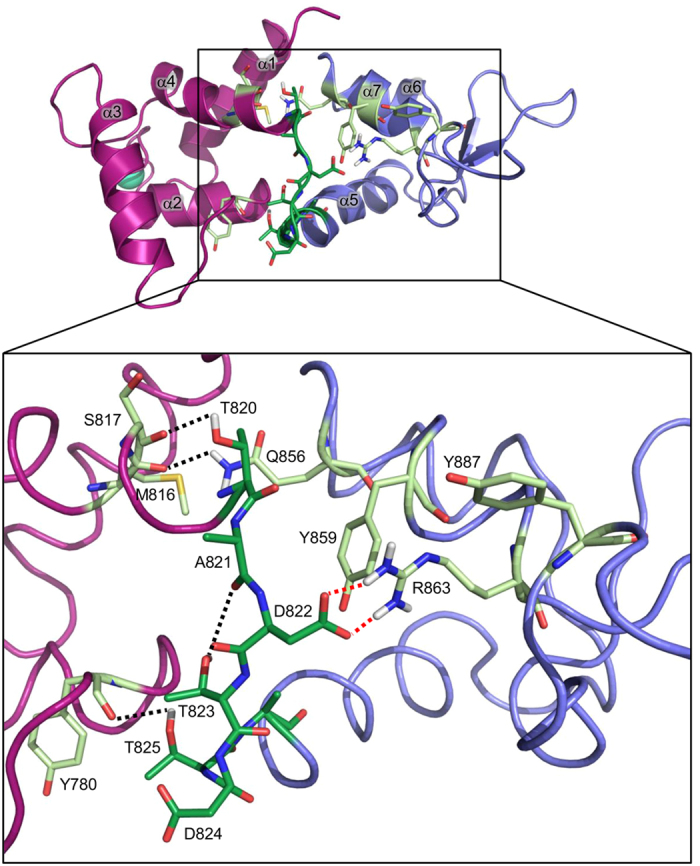
Stabilizing interactions between the linker and the N- and C-terminal lobes in the holo form of CaMD of α-actinin-1. EF1-2 is shown in magenta, EF3-4 in violet, linker between them in dark green, and Ca^2+^ as green-cyan sphere. Residues involved in linker stabilization are shown as light green sticks. Hydrogen bonds and salt bridges are depicted as black and red broken lines, respectively.

In the holo form, the linker is stabilised in position via a hydrogen bond interaction between Thr820 and the main chain carbonyl atom of Ser817 of the preceding α4 helix. Its main chain conformation is additionally stabilised by a hydrogen bond between the carbonyl group of Ala821 and the side chain of Thr823, allowing the side chain of Asp822 to project toward the C-terminal lobe and engage in a salt bridge with Arg863. Arg863 is further stabilised in this position by hydrophobic interactions between its aliphatic side chain and the aromatic rings of Tyr859 and Tyr887 from EF4 helices α7 and α8, respectively (Fig. 5).

Tyr859 is additionally engaged in a hydrophobic interaction, which stabilises the conformation of the Gln856 side chain (helix α7 of EF4), which in turn hydrogen bonds to the main chain carbonyl atom of Met816, residing in helix α4 of EF2; connecting in this way the N- and C-terminal lobes. Furthermore, a linker residue Thr825 forms a short hydrogen bond with the main chain carbonyl atom of Tyr780 (α3 of EF1), further stabilising the inter-lobe orientations (Fig. 5).

### Calcium binding opens EF1-2 of α-actinin-1 CaMD

In order to understand the molecular mechanism causing the above described structural stabilization of CaMD upon calcium binding, we performed a detailed structural comparison of the apo and holo forms of individual EF-pairs of the N- and C-terminal lobes. The extent of structural changes leading to the closed-to-open transition of EFs hands varies for different Ca^2+^ binding proteins. In this context, CaMD of α-actinin-1 would be classified as a protein that undergoes a transition from a closed to a semi-open state (see Supplementary Video).

**Figure 6:**
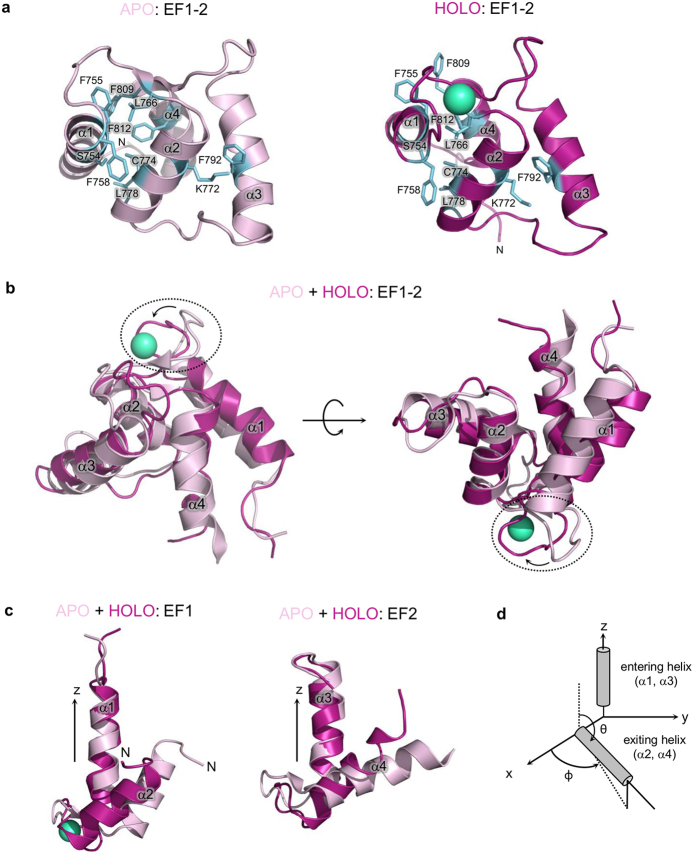
Comparison of EF1-2 of apo and holo forms of CaMD of α-actinin-1. (**a**) Structure of EF1-2 of apo (left) and holo (right) forms of CaMD of α-actinin-1. Calcium ion is shown as a green-cyan sphere. Residues involved in hydrophobic, cation-π and π-π interactions are shown as blue sticks. (**b**) Superposition of EF1-2 of apo and holo forms of CaMD. The most significant conformational changes upon calcium binding are observed at the linker loop between α1 and α2 helices of EF1 (encircled). (**c**) Superposition of separate EF1 and EF2 hands of apo and holo forms of CaMD demonstrate that upon calcium binding significant conformational changes occur within CaMD. Structures of the two forms of EF1 and EF2 were superimposed in the region corresponding to α1 and α3 helix, respectively. (**d**) Schematic display of the vector geometry mapping method (VGM) used to characterize EF motifs in terms of relative interhelical angles. The entering helix (α1, α3) of the EF hand is superimposed on a reference EF hand on the z-axis, and the corresponding position of the exiting helix (α2, α4) is evaluated using the angles θ and ϕ.

In the absence of calcium, three pairs of hydrophobic interactions between residues on helices α1 and α2 provide a tight internal core in EF1: Phe758–Cys774, Phe755–Leu766 and Ser754–Leu778 (Fig. 6a). The same hydrophobic core is present in α-spectrin^22^. In EF2 of CaMD, the corresponding two helices (α3, α4) are further apart from each other, so internal interactions are weaker than in EF1. In EF3-4, the hydrophobic core is formed mostly by residues Phe833 (α5), Leu836 (α5), Ile843 (β), Leu848 (α6), Leu852 (immediately after α6), Ile861 (α7) and Met864, similarly to EF3-4 of α-actinin-2 (ref. 23).

Binding of calcium has a significant impact on the structure of the EF-loops in EF1-2, while the corresponding regions within EF3-4 are largely unchanged. As expected, the majority of movement occurs at EF1, where the calcium-binding loop is completely reorganized (Fig. 6b). Calcium chelating residues are forced to point toward each other and the EF1-2 lobe slightly opens (Fig. 6b,c). The movement breaks the stabilizing interaction Ser754-Leu778, but does not affect the other two hydrophobic interactions. Additionally, a cation-π interaction between Lys772 located in the α2 helix of EF1 and Phe792 from the α3 helix of EF2 is lost (Fig. 6a). Additionally, the side chains of Phe809 and Phe812 are moved further apart (Fig. 6a). Since EF2 lacks a hydrophobic core, it displays a concerted movement with EF1, mediated by hydrophobic interactions between EF1 and EF2, similar to α-spectrin^22^. The short loop between the two helices of both EF1 (α1, α2) and EF2 (α3, α4) motifs has a β-strand conformation in the apo form (Fig. 6a), whereas in the holo form this structural element is absent.

On the other hand, there is little difference between the holo and apo forms of EF hands 3 and 4 in CaMD in α-actinin-1 except for the C-terminal helix (α8), which does not display perfect helical geometry and hydrogen bonding as observed in the apo form. The reason for such subtle changes can be the considerable internal mobility of the helices within the globular domains. This is especially true for the C-terminal domain of calmodulin, which likely exists in a conformational equilibrium between a compact calcium-free structure and a partially open conformation resembling more closely the structure of the calcium-bound state^24^,^25^,^26^.

To quantitatively compare the extent of conformational changes upon Ca^2+^ binding of the EF hands of α-actinin-1, we calculated θ and ϕ angles between entering and exiting helices of EF hands for both apo and holo forms using vector geometry mapping (VGM) (Fig. 6d)^11^ (note that EF4 hand, was excluded from calculations due to its poorly defined exiting helix). Angles were calculated for all structures in an ensemble; mean values were used for comparison and calculation of the angle change (angle_holo_ − angle_apo_) (Table 2). Inter-helical opening due to calcium binding to EF1, as defined by the difference in θ angle (Δθ) which provides a measure of the degree of opening, is largest in EF1. In contrast, in other EF hands the change is less pronounced. On the other hand, the horizontal plane angle (ϕ) change is largest in EF2, while EF1 and EF3 exhibit somewhat smaller changes. In the holo form, the absolute value of θ is largest in EF3, and is comparable with the conformation of EF hands in the bound form.

### NMR chemical shift perturbation confirms a single Ca^2+^ ion binding site

**Figure 7:**
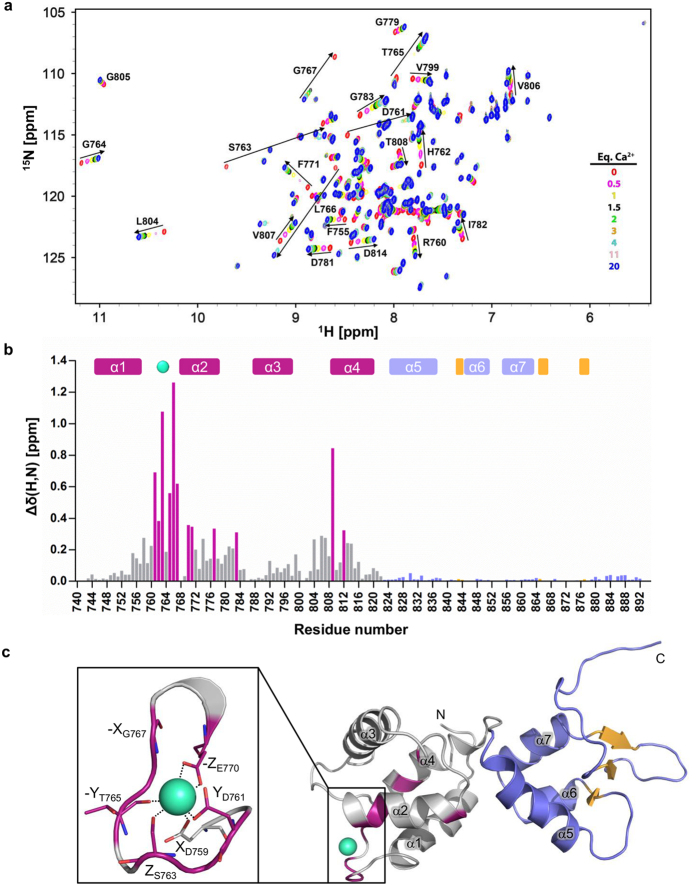
Interaction of CaMD of α-actinin-1 with Ca^2+^ ions. (**a**) Overlay of ^15^N-HSQC spectra of the CaMD of α-actinin-1 during titration with Ca^2+^ ions. Only residues with significant chemical shift changes are labelled. For clarity, only nine out of fifteen titration data points between 0 and 20 eq. of Ca^2+^ ions are presented. (**b**) Chemical shift perturbations of the CaMD of α-actinin-1 in the presence of 20 eq. Ca^2+^ ions. Δδ(H,N) is defined by equation 1. Residues above the threshold value Δδ(H,N) of 0.3 ppm are coloured magenta. α-helical and β-strand secondary structure elements of the holo form are presented as rectangles at the top of the panel. (**c**) CSP of amides mapped on the structure of Ca^2+^-bound CaMD of α-actinin-1. Residues above the threshold value Δδ(H,N) of 0.3 ppm are coloured magenta. The Ca^2+^-coordinating loop of CaMD of α-actinin-1 with coordinating side chain groups of D759, D761, S763, E770, CO group of the T765 main chain and a water molecule hydrogen bonded to G767 are shown enlarged. Ca^2+^ ions are shown as green-cyan spheres.

Calcium binding to EF hand proteins often causes extensive conformational changes in the loop regions of the EF hands, which can be experimentally verified by monitoring changes in NMR chemical shifts. In order to identify the residues affected by calcium binding, we followed chemical shift perturbations of amide resonances in ^15^N-HSQC spectra during titration of CaMD of α-actinin-1 with Ca^2+^ ions, which enabled identification of structural changes and identification of residues involved in Ca^2+^ ion binding (Fig. 7a).

As expected, the chemical shift perturbation (CSP) values observed for the N-terminal lobe (EF1-2) were considerably higher than those observed for the C-terminal lobe (EF3-4). The highest CSPs, with maximum values between 0.3 and 1.24 ppm, are observed for residues that are directly involved in Ca^2+^ binding in EF1 (calculated *K*_d_ of 170 μM). High CSP values of Δδ(H,N) above 0.3 ppm were also observed for a few residues in the loop connecting EF1 and EF2 and in the EF-loop in the EF2 motif (Fig. 7b). Interesting dynamic phenomena were observed as the concentration of Ca^2+^ ions was increased. A few cross-peaks disappeared (Asp761, Thr765, Gly767 and Glu770 during the change from 0.25 to 1.00 equiv. of Ca^2+^ ions), while some cross-peaks (Ser763 and Leu766) could be observed only in the absence, or above 3 molar equiv. of Ca^2+^ ions.

In addition to residues forming the Ca^2+^ ion binding site, significant chemical shift changes (Δδ(H, N) above 0.1 ppm) were also observed for several distant residues. Here, analysis of the NMR structures revealed that these changes correspond to altered stacking patterns of residues with aromatic side chains, which correlate with rearrangements of helices in EF1-2 during Ca^2+^ ion titration.

The above analysis of CSP values as a function of amino acid sequence (Fig. 7b), together with the resonance broadening observed during titration experiments, corroborate our MS and ITC results, and collectively suggest the presence of a single Ca^2+^ ion binding site in the EF-loop of EF1 (Fig. 7c).

## Discussion

In order to contribute to elucidation of the molecular basis of calcium regulation of α-actinin-1 crosslinking of actin filaments, we investigated the effects of calcium binding on α-actinin-1 focusing on its EF hand containing calmodulin-like domain. Using NMR spectroscopy we determined the first high-resolution three-dimensional structures of apo and holo forms of CaMD. Using mass spectrometry and isothermal titration experiments, we found that only one of the four EF hands in CaMD binds calcium, and that binding occurs in an endothermic and enthropically driven manner. This single Ca^2+^ binding site in EF1 of CaMD was mapped by NMR titration experiments. Comparative structural analysis revealed that the apo form displays notable structural flexibility compared to the holo form, and that calcium binding induces local structural changes and contributes to the overall structural rigidity of CaMD.

EF domain containing proteins often display a variable number of “active” EF hand motifs that are able to bind calcium ions, for example certain EF hand motifs in myosin light chain or calpain^27^. Thus, in the present study we first set out to unequivocally determine how many and which EF hand motifs are active in α-actinin-1. Sequence analysis, ESI-MS and ITC experiments conducted on wild-type and mutant CaMD forms (mutations within EF1 and EF2) clearly show only one active Ca^2+^ binding site within EF1. Moreover, dissociation constants from the present study (104.2 ± 15.4 μM) are similar to reported values (56 ± 18 μM)^21^. The discrepancy between these reported values can possibly be attributed to construct differences in the N-terminal region where the construct of α-actinin-1 used in the previous report had an additional stretch of 28 amino acid residues, corresponding to an α-helix from the preceding spectrin domain (SR4). A similar effect has been observed in the case of EF hand of polycystin-2, where the construct with an additional N-terminal stretch of 16 amino acid residues exhibited a 10-fold lower *K*_d_ than the construct encompassing only the EF hand motif^28^,^29^.

The presence of the additional N-terminal helix in CaMD from the previous study^21^ may also account for differences in observed thermodynamic profiles; our shorter construct displays an endothermic binding profile, while the longer construct with the N-terminal helix exhibited an exothermic profile. Still, for both constructs calcium binding appears to be entropically driven due to favourable effects of relieved electrostatic repulsion in the binding loop and increased solvent entropy. While high dehydration enthalpy of bivalent cations significantly contributes to the overall binding energetics, the main contribution corresponds to changes in conformational entropy associated with motion between different structural states^30^ that is linearly related to changes in overall binding entropy^31^. Indeed, our observed thermodynamic profile for CaMD indicates changes in internal dynamics, which can be described as a transition from a disordered or more flexible state to a more structured calcium-bound state^32^. Stabilization of overall structure by Ca^2+^ binding has also been observed for some other proteins, e.g. synaptotagmin^33^,^34^,^35^. Therefore, changes in internal dynamics could have a significant impact on the favourable changes in conformational entropy observed here, e.g. interactions between CaMD with rod domain and with the ABD from the juxtaposed subunit.

In full-length α-actinin-1 the *K*_d_ for calcium binding could be different than observed for the isolated CaMD. For example, it has been reported that there is a 10-fold difference in *K*_d_ for calcium binding by CaMD^BM-40^ (0.8 μM) and full-length BM-40 (0.08 μM). Here, it has been concluded that interdomain contacts significantly contribute to calcium binding via a mutual stabilizing effect, thereby resulting in stronger calcium binding in the case of full-length BM-40 (refs 36,37). Similarly, in S100A4 the presence of the target protein MIIA results in a calcium binding affinity more than one order of magnitude higher than in the absence of this target^38^. Therefore, it is clear that other factors such as the presence of other domains and/or target proteins can significantly affect calcium binding affinity in EF motif containing proteins which could also be the case in α-actinin-1.

Calcium-binding proteins containing EF hand motifs can be classified into two distinct groups depending on the extent of conformational changes upon calcium binding. Members of the first group typically exhibit no or only subtle changes in conformation, in line with their typical function in buffering and/or transport of calcium ions^39^,^40^. In contrast, calcium-dependent conformational changes are much more pronounced in members of the other group. Here, binding of calcium typically triggers a major conformational change in the globular domain from a closed into an open state, causing a rearrangement of the α-helices and exposure of hydrophobic residues on the surface of the protein, allowing the protein to interact with specific partners^41^. The extent of this close-to-open transition in EF hands is different for different proteins, and ranges from large changes (for example, in calmodulin and troponin C) to fairly small changes (calbindin D_9k_)^42^. Analysis of structures of apo and holo forms of α-actinin-1 CaMD, reported in the present study, reveal some conformational changes in EF hands upon calcium binding. However, these changes are not directly analogous to the structural rearrangements observed for other EF hand proteins with similar function (e.g. calmodulin)^16^. In fact, considering the relative orientations of helices in each EF hand motif, the extent of changes reported for CaMD upon calcium binding compared to previously reported interhelical angle changes in calmodulin and troponin C (+26° < Δθ< + 60°, −34° < Δϕ < 0°)^11^ suggest that CaMD of α-actinin-1 undergoes relatively moderate Ca^2+^ induced conformational changes, similar to EF1 of spectrin (Δθ = −1.5°, Δϕ = −46°) (ref. 23). Here, the changes are mostly limited to clockwise swing about the entering helix (characterized by ϕ) while the opening of the helices (characterized by θ) is small compared to the range observed in several other proteins (from −8° to + 60°) (ref. 11).

Regulation of actin-crosslinking proteins, including α-actinin-1, is essential for the normal function and dynamics of the actin cytoskeleton. For α-actinin, plausible models of calcium regulation mechanisms must take into account the vicinity of the neck region of the juxtaposed subunit, which, according to our data, plays a critical role in calcium-regulated rearrangements at the ends of the α-actinin-1 dimer. The important role of the neck region is further highlighted by a recent report on the paralogue α-actinin-2. Here, binding of PIP_2_ to ABD results in release of EF3-4 from the neck region, which unfolds in the absence of EF3-4. This conformational change enables EF3-4 to interact with titin (see Supplementary Fig. S4), while the unfolded neck region ensures flexibility and enables the proper orientation of ABD needed for F-actin binding^14^,^23^. The same basic structural principle has also been reported for the calcium-binding domain of the homologous protein α-spectrin, where EF3-4 bind an α-helical region of the adjacent subunit^43^. Although the mechanism of regulation differs, the high sequence similarity between α-actinin-1 and 2 suggest a similar overall mechanism, where the central feature appears to be binding/release of the neck region by EF3-4. According to our NMR structural data, the calcium stabilized holo form of α-actinin-1 CaMD is more capable of binding the neck region than the apo form, because of the presence of a slightly more open conformation in EF3-4.

**Figure 8:**
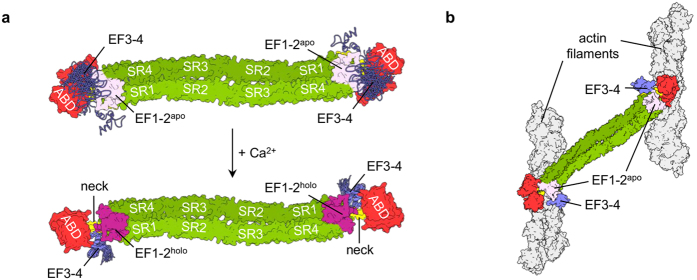
Proposed model of structural changes accompanying Ca^2+^ binding to EF1-2 of α-actinin-1 and subsequent altered affinity for actin filaments. (**a**) Model of the α-actinin-1 dimer depicting ordering of CaMD upon Ca^2+^-binding. For CaMD, NMR ensemble structures superposed in the EF1-2 region were used. Apo and holo forms of EF1-2 are shown in light pink and magenta surface representation, and EF3-4 as violet ribbons. The central feature of the model is increased rigidity in the linker region between the EF1-2 and EF3-4 lobes upon Ca^2+^ binding that could result in stabilization of the interaction between EF3-4 and the neck region. (**b**) Only α-actinin-1 with EF1-2 in the apo form is able to bundle actin filaments. Color coding is the same as in Fig. 1. Models were prepared using NMR structures of CaMD of α-actinin-1 reported in the present study, and structures of human α-actinin-2 (PDB ID 4D1E)^14^ and F-actin (PDB ID 3LUE)^56^.

Considering previous reports on α-actinin-1 as well as results from the present study, we propose the following mechanism of CaMD action in the context of full-length α-actinin-1. In the absence of calcium, CaMD is flexible and either not associated or loosely associated with the neck. Upon calcium binding, the EF3-4 of CaMD wraps around the neck from the adjacent subunit (Fig. 8a). This structural rearrangement consequently locks the ABD of α-actinin-1 in a position that prevents proper relative orientations of ABDs in α-actinin dimer to confer cross-link F-actin in parallel or anti-parallel bundles (Fig. 8b), but does not impede F-actin binding^9^. The proposed mechanism is in agreement with a structural model of α-actinin-4 (ref. 13). However, further experiments considering inter-domain interactions are necessary to unequivocally confirm our proposed model.

From the determined Ca^2+^ binding affinities and the known concentration of calcium in resting non-muscle cells (0.1–1 μM)^44^ it can be concluded that the α-actinin is bound to the actin filaments. Its release is triggered by elevated cytosolic calcium concentrations. While the calcium binding affinity for the EF1 in the context of full-length α-actinin-1 could be much higher (i.e. lower *K*_d_) the model outlined here still remains plausible, especially in the light of known intracellular calcium gradients in migrating cells. Here, lower cytosolic calcium concentration observed at the leading/front side of the cell (in the direction of the movement) would support stabilization of the F-actin bundles within the cell protrusions, while higher cytosolic calcium concentrations at the trailing end would result in destabilization of such bundles and, consequently, destabilization of adhesion structures, essentially allowing the cell to move forward.

Similarly, the effect of calcium binding on interaction of α-actinin-1 with other proteins (i.e. not actin) has not been studied yet, as nicely pointed out in a recent review on non-muscle actinins^5^. It is however known that the vast majority non-muscle α-actinin-binding proteins interact with it *via* its rod domain^1^. This would imply that these interactions are not calcium-dependent (except if those proteins are regulated by calcium, of course). Although the structure of non-muscle α-actinin in calcium-free nor in calcium-bound form is yet unknown we here hypothesise that the calcium-induced conformational changes are largely limited to the ABD-neck-CaMD region since the rod domain appears to be quite rigid.

The high similarity between α-actinin and spectrin family of actin binding proteins allows us to further speculate that this could be a general molecular mechanism for regulating actin crosslinkers.

## Methods

### Cloning and protein expression

A DNA fragment encoding wild-type CaMD (representing exons 18, 19a, 20 and 21) was amplified by PCR using full-length α-actinin-1 cDNA cloned into a modified pET-3d vector as a template. On the 5′-end an N-terminal His_6_-tag, tobacco etch virus (TEV) protease cleavage site and a short spacer (GSS) were introduced. After ligation into the same vector, the construct assembly was verified by sequencing. CaMD mutants (D759A, E770A and D800A) harbouring alanine substitutions within the predicted calcium-binding loops 759−770 and 800−811 were constructed by PCR site-specific mutagenesis (for details see Supplementary Methods). The recombinant proteins were expressed in *E. coli* BL21 (DE3) pLysS. Briefly, cells harbouring the expression plasmid were grown at 37 °C in LB media to an OD_600_ of approximately 0.7. Protein expression was induced by addition of IPTG to a final concentration of 0.5 mM and cells were grown for additional 4 h at 37 °C. After expression, cells were harvested by centrifugation at 6,000 g for 15 min. Cell pellets were resuspended in buffer L (20 mM Tris-HCl pH 7.6, 150 mM NaCl, 10 mM imidazole) and stored at −80 °C.

### Purification of recombinant proteins

Cells were lysed by sonication. After removal of insoluble material by centrifugation the soluble lysate fraction was loaded onto an IMAC column (Ni-NTA agarose, GE Healthcare) pre-equilibrated with buffer A (50 mM Tris-HCl pH 7.6, 500 mM NaCl, 20 mM imidazole). Unbound material was removed by washing with the same buffer and bound protein was eluted with buffer B (50 mM Tris-HCl pH 7.6, 500 mM NaCl, 30 mM imidazole). The N-terminal His_6_-tag was cleaved using His_6_-tagged TEV protease during overnight dialysis against buffer C (50 mM Tris-HCl pH 7.6, 50 mM NaCl, 2 mM β-ME). This resulted in protein containing three additional N-terminal residues (GSS linker). The released N-terminal fragment (His_6_-TEV) and the TEV protease were removed by another IMAC step. The recombinant protein in the flow-through was further purified by size exclusion chromatography using an appropriate buffer (depending on sample preparation for specific methods) using Superdex 75 10/300 column (GE Healthcare). The purity of final samples were verified by SDS-PAGE analysis (see Supplementary Fig. S5).

### Isothermal titration calorimetry

ITC experiments were performed using a MicroITC200 system. Standard ITC conditions were 20 mM HEPES pH 7.6, containing 100 mM NaCl at 25 °C. All samples were treated with Chelex^®^ 100. In some measurements 1.5 mM DTT was also included. For a typical Ca^2+^ titration, wild-type or mutant CaMD protein solution, at concentrations ranging from 0.26 to 0.72 mM, was placed in the reaction cell. A solution of calcium chloride (5–20 mM) was loaded into the syringe, and then 2 or 4 μl of Ca^2+^ solution was injected into the reaction cell, usually at 5 minute intervals. Blank injections of titrant into the corresponding buffer were used to account for heat of mixing and dilutions. To obtain the thermodynamic parameters ΔH, ΔS and ΔG, injection profiles were analysed using the single-site model of Sedphat^45^.

### Native electrospray ionization mass spectrometry

Wild-type and mutant CaMD samples for ESI-MS analysis were prepared as described previously in the expression and purification protocol. However, the final SEC purification step was performed using 10 mM ammonium acetate pH 7.6, 2 mM calcium acetate and 1 mM DTT. Following buffer exchange (10 mM ammonium acetate pH 8.2), samples were further diluted 1:10 in 10% methanol, transferred to borosilicate emitter tips (Thermo Fisher Scientific) and directly infused into an Orbitrap Velos Pro mass spectrometer (Thermo Fisher Scientific) using a source voltage of 1–1.6 kV. Spectra were recorded at a resolution of 60,000 at m/z 400 with 10 microscans at an AGC target value of 1E6, applying an in-source fragmentation of up to 80 kV. Spectra were averaged and charge states +9 up to +14 were deconvoluted using the Xtract software package (Thermo Fisher Scientific) to determine monoisotopic masses.

### NMR spectroscopy

For NMR spectroscopy, ^13^C- and ^15^N-isotopically labelled CaMD of α-actinin-1 was prepared by expression in M9 minimal growth medium^46^ supplemented with ^13^C D-glucose and ^15^NH_4_Cl as the carbon and nitrogen source, respectively.

All NMR experiments were performed on a Varian 800 MHz NMR spectrometer equipped with a triple ^1^H/^13^C/^15^N resonance cryogenic probe head with inverse detection at 298 K. Spectra were recorded in the absence (buffer 20 mM HEPES pH 7.6, 100 mM NaCl, 50 μM EGTA, 1.5 mM DTT) and presence of Ca^2+^ ions (buffer 20 mM HEPES pH 7.6, 100 mM NaCl, 2 mM CaCl_2_, 1.5 mM DTT). Protein concentrations were 1 mM. For titration, calcium chloride was added stepwise to a final concentration of 20 mM. NMR experiments with detection of HN were performed in 90%/10% H_2_O/D_2_O, pH 7.6 and for detection of HC in 100% deuterated buffer. For assignments, standard double and triple resonance NMR experiments were performed. Assignments were followed by determination of sequential, medium-range and long-range NOEs restraints in the 3D ^15^N-edited NOESY-HSQC experiment. ^1^H and ^13^C resonances of aliphatic and aromatic side chains were assigned using HAHB(CO)NH, CC(CO)NH, (H)CCH-TOCSY and ^13^C-edited NOESY-HSQC 3D experiments (for details see Supplementary Data). For Ca^2+^ ion titration experiments, the protein (final concentration of 0.312 mM) and buffer (20 mM HEPES pH 7.6, 100 mM NaCl, 1.5 mM DTT) were pre-treated with Chelex^®^ 100 to completely remove traces of Ca^2+^ ions. All spectra were processed by NMRPipe^47^ and analysed with CARA^48^ and Sparky^49^.

### Structure calculation

Initial structure calculations were performed using CYANA 3.0 (ref. 50). A high-resolution structure of the apo form was obtained using 1,364 intra- and sequential, 768 medium-range and 591 long-range distance constraints, supported by 214 backbone torsion angle restraints followed by final refinement using RDC restraints. For the holo form, 1,226 intra- and sequential, 711 short-range and 675 long-range distance constraints supported by 208 backbone torsion angle restraints were used and the final structure was refined using RDC restraints (see Supplementary Table S1). Structure refinements for both forms were performed using an explicit solvent model in YASARA^51^. All structure figures were prepared in PyMOL (Schroedinger) except those in Fig. 8 that were prepared in Chimera^52^.

### Chemical shift perturbation (CSP)

Amide chemical shift changes (Δδ) or chemical shift perturbations were calculated for each non-overlapping residue in ^15^N-HSQC spectrum according to equations (1) and (2):


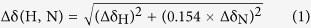



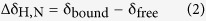


where Δδ_H_ and Δδ_N_ are the ^1^H and ^15^N amide chemical shift changes induced by calcium binding and the resulting structural changes. CSPs were defined as the difference between the corresponding chemical shifts in the bound and free states of the CaMD^53^.

### Dissociation constant determination from CSPs

Titration data were analysed assuming that the observed chemical shift perturbation Δδ is the weighted average between the two extreme values corresponding to the free (Δδ = 0) and ligand-bound states (Δδ = Δδ_obs_). CSPs data were nonlinearly fit against the theoretical 1:1 model of protein-ligand interaction using the simple binding equilibrium:


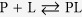


where P stands for protein, L for ligand (Ca^2+^) and PL for the protein-ligand complex. The equilibrium dissociation constant *K*_d_ is defined as in equation (3):


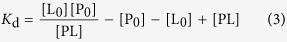


where [P_0_] and [L_0_] are total concentration of protein and ligand and [PL] the concentration of protein-ligand complex at equilibrium. A 1:1 model with one calcium ion bound to the CaMD gives:


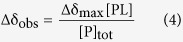


Equations (3) and (4) yield equation (5):


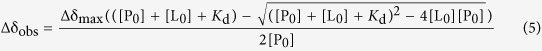


where Δδ_obs_ is the change in observed chemical shift from the free state, and Δδ_max_ is the maximum chemical shift change at saturation. Data were fit using Origin 8.1 (Origin Lab).

## Additional Information

**Accession codes:** The structural ensemble has been deposited in the PDB under ID code 2N8Z and 2N8Y for the apo and holo forms of CaMD, respectively.

**How to cite this article**: Drmota Prebil, S. *et al.* Structure and calcium-binding studies of calmodulin-like domain of human non-muscle α-actinin-1. *Sci. Rep.* **6**, 27383; doi: 10.1038/srep27383 (2016).

## Supplementary Material

Supplementary Movie 1

Supplementary Information

## Figures and Tables

**Figure 1:**
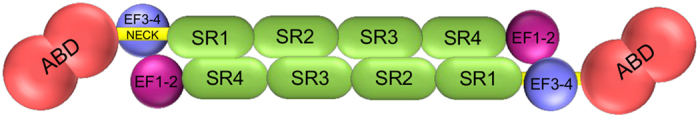
Domain structure of α-actinin-1. Domain composition of α-actinin antiparallel dimer, as inspired by the structure of human α-actinin-2 (ref. 11). ABD is shown in red, neck peptide in yellow, SR1−SR4 in green, EF1-2 in magenta and EF3-4 in violet.

**Table 1:** Thermodynamic parameters of calcium binding to wild-type and CaMD_D800A mutant obtained by ITC.

Construct	*K*_d_	n	ΔH	TΔS	ΔG
	μM		kcal/mol	kcal/mol	kcal/mol
CaMD_wt	104.2 ± 15.4	0.75 ± 0.07	2.10 ± 0.12	7.54 ± 0.17	−5.44
CaMD_D800A	82.2 ± 12.9	0.84 ± 0.05	2.05 ± 0.20	7.63 ± 0.19	−5.58

Values correspond to the mean of seven experiments for CaMD_wt and eight experiments for CaMD_D800A. Data for mutants CaMD_D759A and CaMD_E770A are not shown since no heat was released or absorbed upon CaCl_2_ injections. Errors are shown as standard deviation.

**Table 2:** Quantitative analysis of conformational changes upon Ca^2+^ binding to CaMD of α-actinin-1.

	θ (absolute value) (°)	Δθ (°)	Δϕ (°)
EF1	+56.3	+15.1	−27.6
EF2	+50.1	−8.7	−35.7
EF3	+62.3	−2.4	−20.0
