# Supplementary material for: Structure and calcium-binding studies of calmodulin-like domain of human non-muscle α-actinin-1
Source: Sci Rep. 2016 Jun 7;6:27383. doi: 10.1038/srep27383 (PMC4895382; doi:10.1038/srep27383)
Supplement: Supplementary Information [file srep27383-s2.pdf]

Structure and calcium-binding studies of calmodulin-like domain of human non-muscle α-actinin-1

Sara Drmota Prebil, Urška Slapšak, Miha Pavšič, Gregor Ilc, Vid Puž, Euripedes de Almeida Ribeiro, Dorothea Anrather, Markus Hartl, Lars Backman, Janez Plavec, Brigita Lenarčič* & Kristina Djinović-Carugo*

Supplementary Methods. Cloning of the constructs.

For preparation of the construct encoding for human wild-type calmodulin-like domain (CaMD) of α-actinin-1, the following pair of oligonucleotides was used (introduced restriction sites *Xho*I and *Mlu*I are underlined):

wtCaMD forward: 5`-CAGGGCTCGAGCATCAGCCAGGAGCAGATGAATGAGTTC (*Xho*I)
wtCaMD reverse: 5`-CTCACGCGTGGATTAGAGGTCACTCTC (*Mlu*I)

CaMD mutants harbouring alanine substitutions within the predicted calcium-binding motif between amino acids 759-770 and 800-811 were created by site-specific PCR mutagenesis using the QuikChange II Site-Directed Mutagenesis Kit (Agilent Technologies). All three mutants (CaMD_D759A, CaMD_E770A and CaMD_D800A) were produced using the wild-type CaMD cDNA as template and the following oligonucleotides primers:

CaMD_D759A forward: 5`-TCCTTCAACCACTTTGCCCGGGATCACTCCG-3`
CaMD_D759A reverse: 5`-CGGAGTGATCCCGGGCAAAGTGGTTGAAGGA-3`
CaMD_E770A forward: 5`-CTGGGTCCCGAGGCGTTCAAAGCCTGC-3`
CaMD_E770A reverse: 5`-GCAGGCTTTGAACGCCTCGGGACCCAG-3`
CaMD_D800A forward: 5`-CATGAGCATTGTGGCCCCCAACCGCCTGG-3`
CaMD_D800A reverse: 5`-CCAGGCGGTTGGGGGCCACAATGCTCATG-3`

Supplementary Data. Sequence-specific NMR resonance assignment.

Sequence-specific assignments of backbone resonances for the apo form were obtained using standard triple resonance NMR experiments (HNCO, HN(CO)CA, HNCA, HN(CO)CACB, HNCACB). NOE distance restraints were determined with the use of 3D ^{15}N-edited and ^{13}C-edited NOESY-HSQC experiments. An almost complete resonance assignment was obtained for each backbone nucleus of the apo form with the exception of Gly(-3), Ser(-2), and Ser(-1) from the N-terminal GSS linker, and Thr868 for which the ^{1}H, ^{15}N cross peak could not be observed, presumably due to dynamics. The overall completeness of resonance assignments including side chains was 97.4%. ^{1}H, ^{15}N, ^{13}C resonances of the holo form were assigned employing the same set of NMR experiments as for the apo form. Gly(-3), Ser(-2), Ser(-1), Thr868 and Gly869 backbone resonances of the holo form could not be identified in the ^{15}N-HSQC spectra. The overall completeness of resonance assignments including side chains was 98.8%.

Supplementary Table S1. NMR restraints and structural statistics for the ensemble of 20 lowest energy structures of apo and holo form of CaMD.

**Restraints**	**apo**	**holo**
NOE upper distance restraints[a]	2,723	2,612
Intra-residue (|i-j|=0)	636	560
Sequential (|i-j|=1)	728	666
Medium-range (1<|i-j|<5)	768	711
Long-range (|i-j|>=5)	591	675
Torsion angles restraints[a]		
Backbone (ϕ/ψ)	214	208
RDC	102	81
RMSD to the mean co-ordinates (Å)		
Ordered backbone atoms	(1-81) 0.65 +/- 0.21<br>(87-15) 0.94 +/- 0.14	(1-153) 1.13 +/- 0.25
Ordered heavy atoms	(1-81) 1.13 +/- 0.42<br>(87-153) 1.46 +/- 0.38	(1-153) 1.37 +/- 0.24
**Structural statistics**		
Ramachandran plot[b]		
Residues in most favoured regions (%)	90.9	90.7
Residues in additionally allowed regions (%)	9.0	8.5
Residues in generously allowed regions (%)	0.1	0.7
Residues in disallowed regions (%)	0.0	0.1
Structure z-scores[b]		
1st generation packing quality	1.047 +/- 0.631	-0.262 +/- 0.499
2nd generation packing quality	4.467 +/- 1.562	3.524 +/- 1.511
Ramachandran plot appearance	-2.776 +/- 0.234	-3.467 +/- 0.277
chi-1/chi-2 rotamer normality	-5.379 +/- 0.309	-6.038 +/- 0.351
Backbone conformation	-0.800 +/- 0.248	-1.355 +/- 0.224
RMS z-scores[b]		
Bond lengths	1.162 +/- 0.010	1.178 +/- 0.025
Bond angles	0.471 +/- 0.015	0.533 +/- 0.047
Omega angle restraints	0.509 +/- 0.039	0.601 +/- 0.060
Side chain planarity	0.469 +/- 0.047	0.990 +/- 0.499
Improper dihedral distribution	0.601 +/- 0.026	0.842 +/- 0.174
Inside/Outside distribution	1.039 +/- 0.015	1.052 +/- 0.010

[a] None of the 20 structures exhibit distance violations over 0.2 Å and torsion angle violations over 5°.

[b] Ensemble of structures was analysed by PROCHECK-NMR and WhatIF programs incorporated in CING structure evaluation package[1].

Supplementary Figures

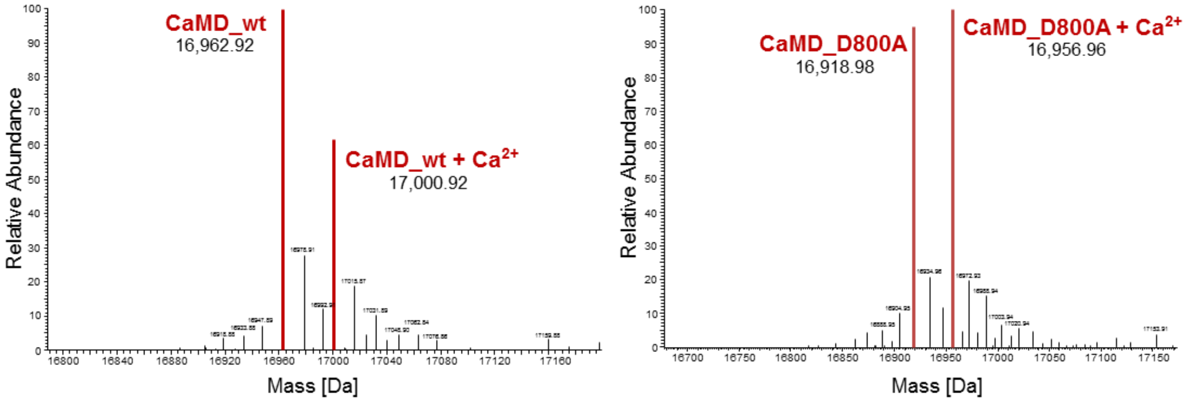

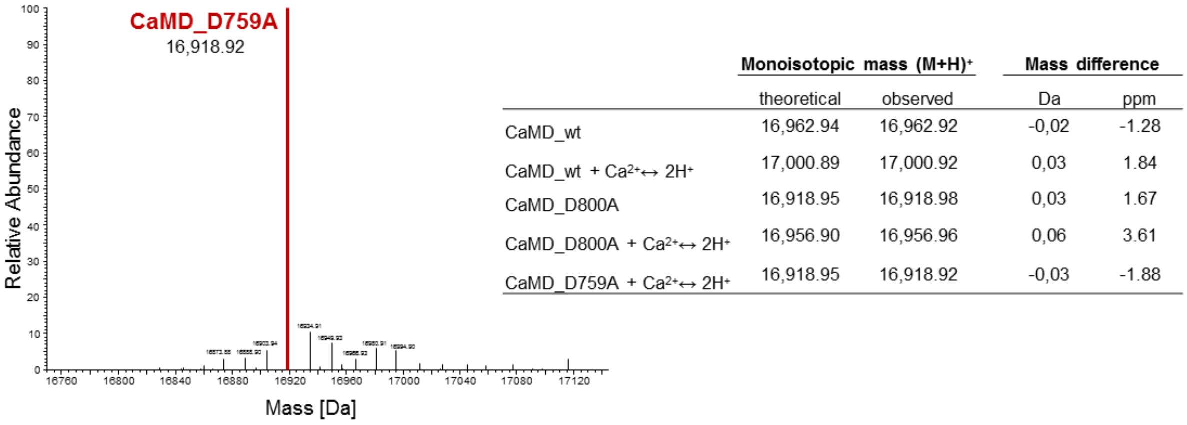

	Monoisotopic mass (M+H)+		Mass difference	
	theoretical	observed	Da	ppm
CaMD_wt	16,962.94	16,962.92	-0,02	-1.28
CaMD_wt + Ca^{2+}↔ $2H^+$	17,000.89	17,000.92	0,03	1.84
CaMD_D800A	16,918.95	16,918.98	0,03	1.67
CaMD_D800A + Ca^{2+}↔ $2H^+$	16,956.90	16,956.96	0,06	3.61
CaMD_D759A + Ca^{2+}↔ $2H^+$	16,918.95	16,918.92	-0,03	-1.88

Supplementary Figure S1. Native ESI-MS: Comparison of deconvoluted mass spectra of wild-type CaMD and its mutants.
The charge states +9 to +14 were deconvoluted to obtain monoisotopic masses ($[M+H]^+$) for all major signals. In wild-type and mutant D800A the two most intense signals corresponded very well to the masses of the molecular ion and its adduct with a single Ca^{2+} ion. For mutant D759A only a signal matching the molecular ion but no calcium adducts were observed. Minor peaks represent oxidation products (e.g. at 16,978.91), salt adducts, or background signals. Theoretical and observed masses of wild-type CaMD and its mutants are represented in the table.

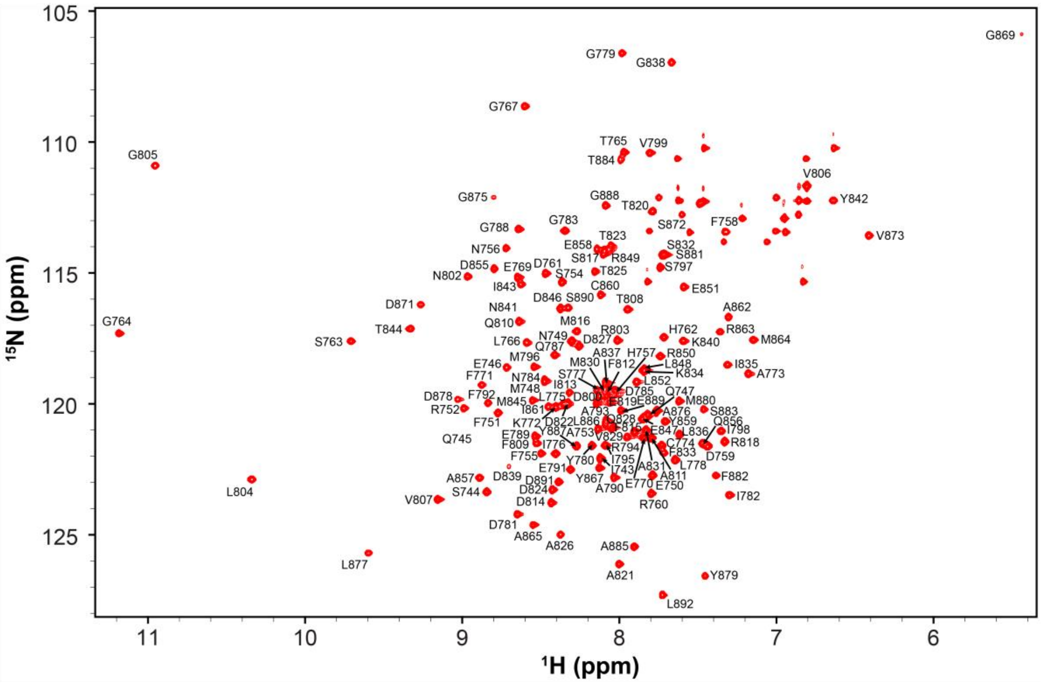

Supplementary Figure S2. ^{15}N-HSQC spectrum of CaMD in the absence of Ca^{2+} ions with one letter amino acid code.

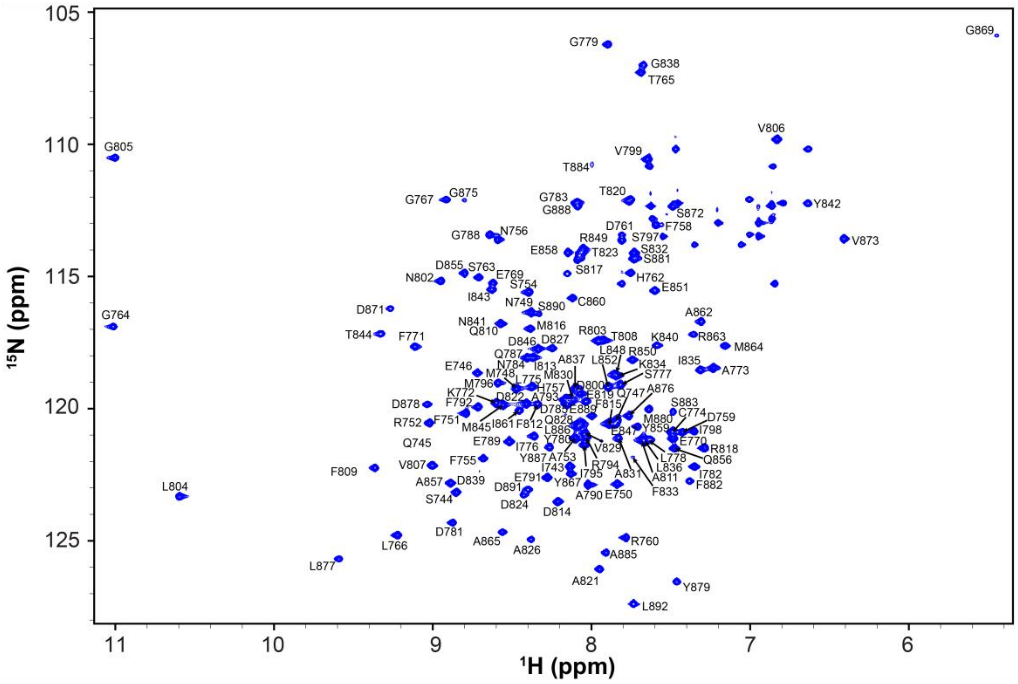

Supplementary Figure S3. ^{15}N-HSQC spectrum of CaMD upon addition of 20 equiv. of Ca^{2+} ions with one letter amino acid code.

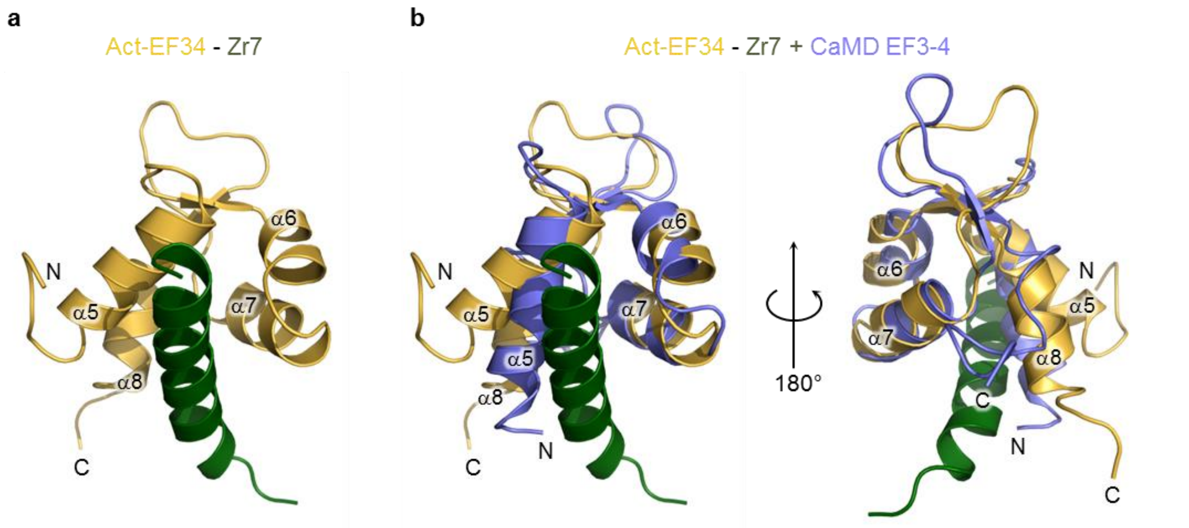

Supplementary Figure S4. Comparison of α-actinin 2-titin Z-repeat complex with EF3-4 from holo form of CaMD of α-actinin 1. (**a**) Structure of the complex of α-actinin 2 EF3-4 (Act-EF34) with titin Z-repeat (Zr7). α-actinin 2 EF3-4 is shown in gold and Zr7 in green (PDB ID 1H8B). (**b**) Superposition of the complex of Act-EF34-Zr7 and EF3-4 of the holo form of CaMD.

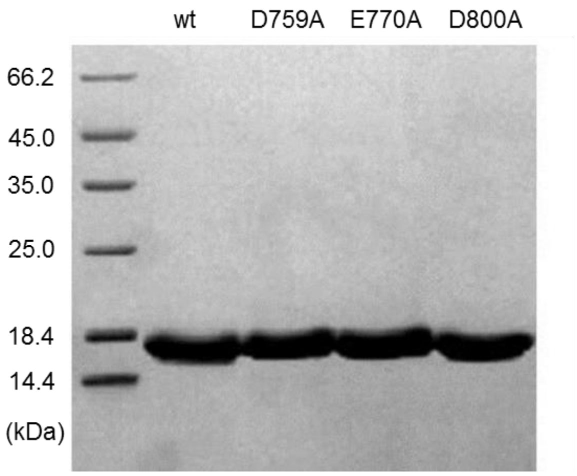

Supplementary Figure S5. SDS-PAGE analysis of wild-type CaMD in its mutants (D759A, E770A and D800A). Analysis of the final preparation steps under reducing conditions. Samples were run on a 15% polyacrylamide gel and were stained with Coomassie Brilliant Blue R-250. Positions of calibrating proteins are given in kDa.

Supplementary Video

Morph of apo-to-holo transition (and back) of a-actinin-1 CaMD triggered by binding of calcium ion to EF1. CaMD is shown as cartoon; EF1-2 is shown in light pink (apo) or magenta (holo), EF3-4 in violet, and the linker region between the two lobes in green. Calcium ion is depicted as a green-cyan sphere, and coordination bonds as yellow sticks. Morph was created using Chimera[2].

Reference

1. Doreleijers, J. F. *et al.* CING: an integrated residue-based structure validation program suite. *J. Biomol. NMR* **54,** 267–283 (2012).
2. Pettersen, E. F. *et al.* UCSF Chimera--a visualization system for exploratory research and analysis. *J. Comput. Chem.* **25,** 1605–1612 (2004).
